# Supplementary figures and images for: Resurgent Na+ Current Offers Noise Modulation in Bursting Neurons
Source: PLoS Comput Biol. 2019 Jun 21;15(6):e1007154. doi: 10.1371/journal.pcbi.1007154 (PMC6608983; doi:10.1371/journal.pcbi.1007154)

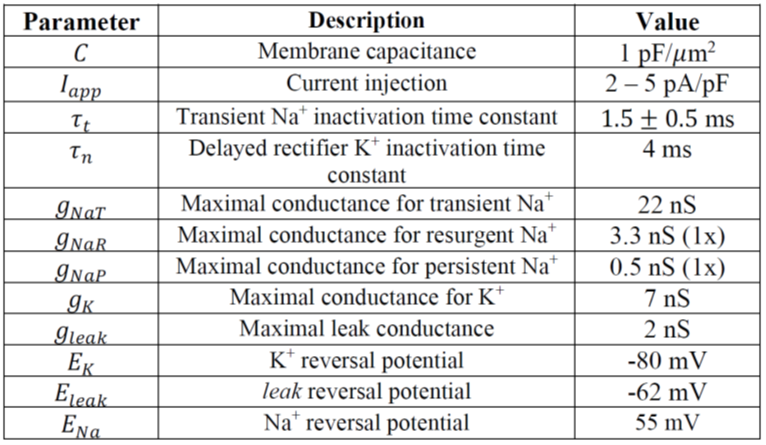

Supplement: S1 Table — (TIF) [file pcbi.1007154.s001.tif]

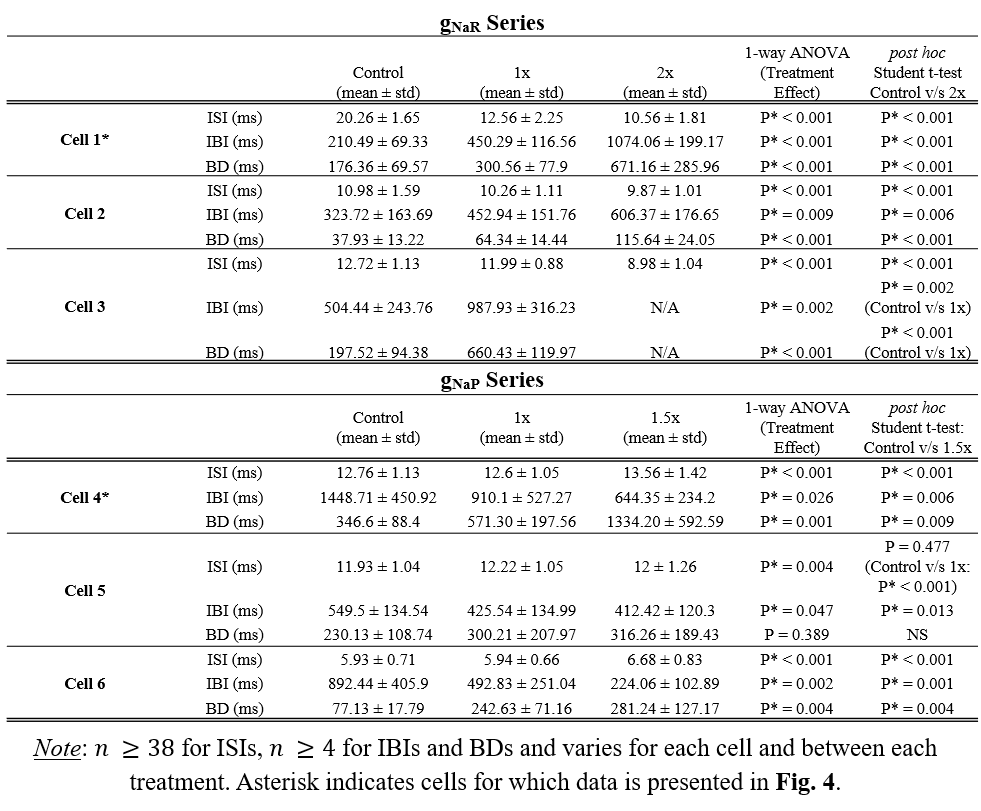

Supplement: S2 Table — (TIF) [file pcbi.1007154.s002.tif]
