# Supplementary material for: Resurgent Na+ Current Offers Noise Modulation in Bursting Neurons
Source: PLoS Comput Biol. 2019 Jun 21;15(6):e1007154. doi: 10.1371/journal.pcbi.1007154 (PMC6608983; doi:10.1371/journal.pcbi.1007154)
Supplement: S1 Text — (DOCX) [file pcbi.1007154.s007.docx]

**S1 Text. Bifurcation Analysis of Model Behavior**

Mathematical analytical approaches enable examination of experimentally intractable mechanisms and provide deeper insights into the behavior of complex nonlinear dynamical systems. We combined model simulations with geometric dynamical systems methods to examine the $I_{NaR}$’s mechanism of burst control. In this supplementary text, we explain the theoretical concepts underlying the so-called *bifurcation diagrams* presented in **Figs. 6d** and **e**. First, to generate theses diagrams, we dissected the system of equations representing the bursting neuron model into fast and slow subsystems^2^. This enabled understanding the qualitative changes in the behavior of fast subsystem consisting of the membrane potential (𝑉) due to changes in the slow kinetics of persistent Na^+^ inactivation/recovery ($h_{p}$) (see **Figs. 6a-c** highlighting relative timescales of 𝑉 and $h_{p}$ during bursting activity). Note that during a burst of activity, $h_{p}$ is inactivating (decreasing) during each spike, however, recovers from inactivation on a relatively slow timescale. In the diagrams in **Figs. 6d** and **e**, we treat the slow variable $h_{p}$ as a parameter that when slowly changes, causes qualitative changes in the fast variable, 𝑉. For example, we can determine for which values of $h_{p}$ the fast subsystem generates either near rest behavior or quiescence (stable equilibria) or spikes (stable oscillations) or both. Note that there are two values of $h_{p}$, $h_{HB}$ (onset threshold) and $h_{SNP}$ (offset threshold), such that 𝑉 exhibits: i) a near rest behavior (stable equilibrium – red curve) for $h_{p}< h_{HB}$; ii) an unstable equilibrium for $h_{p}> h_{HB}$; and iii) spiking for $h_{p}< h_{SNP}$ (stable oscillations – green circles). As $h_{p}$ gradually increases during IBI, indicating channel recovery, the membrane potential, 𝑉 transitions from a resting state (or stable equilibria) until it reaches the Andronov-Hopf bifurcation point, labeled “onset threshold” (e.g., **Figs. 6d**), that is representative of spike onset threshold. Past this threshold, 𝑉 becomes unstable and spiking phase begins whose amplitudes are bounded by the upper and lower green curves marking the maximum (peak) and minimum (trough) values of membrane potential, 𝑉. These represent stable oscillations (or periodic solution) when the neuron is in the bursting phase. For, $h_{SNP}< h_{p}< h_{HB}$, the membrane potential, 𝑉 shows bistability: for those values of $h_{p}$, there is both a stable equilibrium and a stable oscillation. The same diagrams are reproduced for specific $g_{NaR}$ and $g_{NaP}$ values in **Figs. 7d-f** where the green shaded region that represents a region of attraction for the stable curve of equilibria are highlighted. Physiologically, this region houses the sub-threshold oscillations that decay in amplitude as a burst terminates.
